# The role of human outdoor recreation in shaping patterns of grizzly bear-black bear co-occurrence

**DOI:** 10.1371/journal.pone.0191730

**Published:** 2018-02-01

**Authors:** Andrew Ladle, Robin Steenweg, Brenda Shepherd, Mark S. Boyce

**Affiliations:** 1 Department of Biological Sciences, University of Alberta, Edmonton, Alberta, Canada; 2 Wildlife Biology Program, College of Forestry and Conservation, University of Montana, Missoula, Montana, United States of America; 3 Parks Canada, Jasper, Alberta, Canada; University of Hyogo, JAPAN

## Abstract

Species’ distributions are influenced by a combination of landscape variables and biotic interactions with other species, including people. Grizzly bears and black bears are sympatric, competing omnivores that also share habitats with human recreationists. By adapting models for multi-species occupancy analysis, we analyzed trail camera data from 192 trail camera locations in and around Jasper National Park, Canada to estimate grizzly bear and black bear occurrence and intensity of trail use. We documented (a) occurrence of grizzly bears and black bears relative to habitat variables (b) occurrence and intensity of use relative to competing bear species and motorised and non-motorised recreational activity, and (c) temporal overlap in activity patterns among the two bear species and recreationists. Grizzly bears were spatially separated from black bears, selecting higher elevations and locations farther from roads. Both species co-occurred with motorised and non-motorised recreation, however, grizzly bears reduced their intensity of use of sites with motorised recreation present. Black bears showed higher temporal activity overlap with recreational activity than grizzly bears, however differences in bear daily activity patterns between sites with and without motorised and non-motorised recreation were not significant. Reduced intensity of use by grizzly bears of sites where motorised recreation was present is a concern given off-road recreation is becoming increasingly popular in North America, and can negatively influence grizzly bear recovery by reducing foraging opportunities near or on trails. Camera traps and multi-species occurrence models offer non-invasive methods for identifying how habitat use by animals changes relative to sympatric species, including humans. These conclusions emphasise the need for integrated land-use planning, access management, and grizzly bear conservation efforts to consider the implications of continued access for motorised recreation in areas occupied by grizzly bears.

## Introduction

Interspecific interactions play a fundamental role in shaping species’ distributions and behavior [1], however incorporating such relationships into species distribution models can be logistically challenging and complex [2]. Interspecific competition can lead to resource partitioning that allows multiple species that are ecologically similar to coexist on the same landscape [3,4], resulting in sympatry with niche divergence [5]. These interactions can be altered by anthropogenic factors such as human activity [6], which has the potential to affect individual behavior and habitat use, both key considerations in management and conservation planning [7]. To understand species distributions and abundance it is important to incorporate such relationships, and factors that influence them [8]. Ignoring biotic interactions, between prey, competitors and predators, can lead to biased or inaccurate inferences regarding an animal’s habitat selection or changes in behavior [9].

Grizzly bears (*Ursus arctos)* and black bears (*U*. *americanus*) are sympatric across the majority of the grizzly bear range in North America, and inhabit similar niches in terms of food preference [10]. Where these two species are sympatric, diet overlap is high; both are opportunistic omnivores [11,12] that rely on berry crops to gain the necessary weight for denning [13,14]. Reliance on the same food source leads to competition, and studies have shown that spatial displacement is common [15]. Altered activity patterns also have been documented, with black bears switching daily activity patterns to reduce overlap with grizzly bears [10]. Although displacement in these examples is due to competition for resources, there have been instances of intraguild predation, exacerbating black bear avoidance of grizzly bears [10]. Conversely, there is little evidence that grizzly bears are negatively affected by black bear occurrence directly, however it is postulated that high black bear densities might result in reduced reproduction by grizzly bears through exploitation competition, despite grizzly bears being able to dominate high-quality foraging through resource defense competition and direct interference competition [12,16].

Human recreational activity is an increasing issue for wildlife [17] and has the potential to affect ecological communities through redistribution and changes in activity patterns [18], and human access to grizzly bear habitat has been highlighted as a key concern moving forward with grizzly bear recovery in Alberta, Canada [19]. Wildlife display differing responses to motorised and non-motorised activity [20,21], which has in part led to restricted motorised recreation in many protected areas. The influence of recreational activity on bear habitat use and behavior could be as important a factor as the interaction between grizzly and black bears, as shown in other species [22]. Many carnivore species that have few or no predators display negative responses to human disturbance [23,24]. Perceived predation risk can have implications for individuals within a population, through spatial displacement [25], temporal displacement [6], changes in movement behavior [26] and increased stress responses [27]. Varying responses to human activity due to different fear perceptions and costs associated with avoidance [28] has led to the ‘human shields’ hypothesis, by which animals use human activity to protect against predation [29]. Both grizzly bears and black bears are influenced by human activity, however this response varies between the two species. Black bears show increased activity near human developments [10]. Grizzly bears in contrast avoid human infrastructure, such as high traffic-volume roads [30,31] and alter their activity patterns to minimise temporal overlap [26].

Occupancy modelling is a statistical framework that allows researchers to investigate the relationship between the presence-absence of a species and associated habitat characteristics. Current methods for modelling occupancy were developed to account for imperfect detection [32]. These models were originally designed for discrete, patch-based occupancy studies, such as territorial birds or amphibians in discrete ponds [33]. Although occupancy software also has been applied to free-ranging animals in continuous habitats [6,34], these applications are not dealing with strict “occupancy” at a site; rather instantaneous occurrence or habitat use of that site [35]. Although occurrence informs us on species distribution across a landscape, and variables influencing this, we lose information by condensing count data to a binary response variable. However, these count data can be used as an informative measure of relative intensity of use, with a value of 1 as high use of a specific camera site. Thus, the “detection probability” in occupancy context is actually a metric of the intensity of use for applications to camera-trap data. In this context, intensity of use is a variable of interest, rather than a nuisance parameter [36]. Occurrence (ψ) is influenced by a population’s distribution across the landscape, representing areas that are used versus areas that are never used. Measures of intensity of use (*p*), for trail camera data is primarily a consequence of variation in population density and individual movement patterns [35,37–39] rather than the ability to detect individuals at a given sample location as in occupancy studies [37]. Both parameter estimates are effected by habitat variables and interspecific interactions with wildlife species and human activity, but infer different scales in terms of the response [40]. Further advances in occupancy modeling have included estimating the probabilities of co-occurrence between two or more species, and how the presence of a species might influence not just the probability of other species’ occurrence, but also the detectability, or intensity of use in the present context, of other species’ [2]. Such models have been applied to questions specific to community dynamics in sympatric owls [41], Madagascan carnivores [42], rails [43] and treefrogs [44]. More recently, multispecies models have been developed that allow comparison of occurrence for two or more interacting species [9], which opens up the ability to test hypotheses relating to community level spatial distribution and habitat use, whilst accounting for habitat preferences.

To evaluate the relative importance of interspecific interactions between two bear species; grizzly bear and black bear, and two types of human recreational activity; motorised and non-motorised, we placed camera traps on human-use trails within Jasper National Park and an adjoining area along the eastern Rocky Mountain foothills of Alberta. We studied changes in occurrence and intensity of use using a suite of habitat variables, presence or absence of sympatric bear species, and presence or absence of recreational activities. In addition, we compared daily activity patterns between pairs of species’ and recreational activities to answer the following questions: 1) To what degree do grizzly bears and black bears occur in different habitats? 2) Do we observe co-occurrence of grizzly and black bears on trails, or are they spatially and temporally separated? 3) How does motorised and non-motorised recreation influence trail use by and activity patterns of grizzly and black bears? and 4) Do we see a relative difference in temporal patterns of bear activity in the presence or absence of motorised and non-motorised recreational activity and competing bear species?

## Methods

All research was approved through Human Ethics Approval at the Research Ethics Office of University of Alberta, project no PRO 00040029 and was covered under the Parks Canada research and collection permit BAN-2012-11113.

### Study area

**Fig 1:**
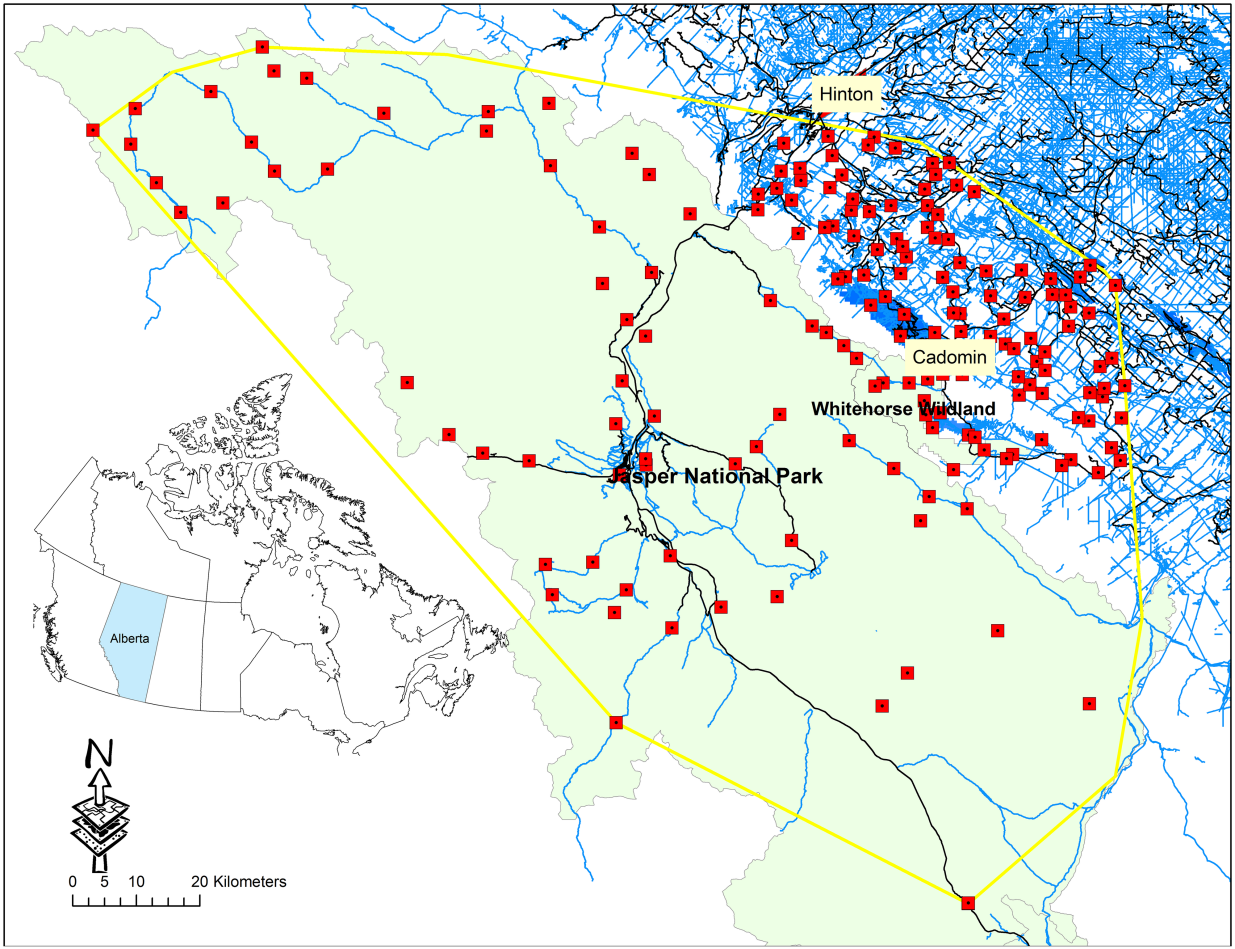
Map of study area in West-central Alberta’s Rocky Mountains and foothills, displaying all 194 camera locations that were active between June 15 and August 25 2014. Red squares represent camera locations. Roads (black) and trails (blue) are displayed, and green shading represents protected areas, including Jasper National Park and Whitehorse Wildland Park.

The study was conducted in the central Alberta’s Rocky Mountains and foothills (Fig 1). The landscape consists of higher elevation, mountainous terrain in the west, and foothills at lower elevations to the east. Forest cover is prominent, and consisting of spruce (*Picea* spp.), fir (*Abies* spp.) lodgepole pine (*Pinus contorta*), aspen (*Populus tremuloides*) and balsam poplar (*P*. *balsamifera*). The study region includes Jasper National Park and Whitehorse Wildland Park, where motorised recreation is not allowed, and public lands with limited restrictions of recreational activity, where motorised recreation is prevalent [45]. Industrial activity, including oil and gas extraction, open-pit coal mining and timber extraction, are present within the public lands to the east, and have contributed to the high density of linear features.

### Trail camera sampling

Trail camera data was used from a combination of two concurrent camera trap studies (see [45,46]for specific details on sampling design). In both studies, we placed cameras on anthropogenic trails using a systematic design, and deployed them more than one km from the nearest other camera locations. We used data collected between June 15 and August 25 2014, because these dates were outside of the black bear hunting season for the region (albertaregulations.ca). We set cameras (Reconyx Inc, Holmen, WI, USA) at 194 trail locations (Fig 1). We monitored sites for > 20 days each. We set cameras to take pictures 24 hours a day at high sensitivity, and took a set of 3–5 pictures in rapid succession when triggered with no delay. At each location, we placed cameras at an angle to the trail and approximately 1-3m from the trail to minimise the probability of missing fast-moving objects such as Off-Highway Vehicles’ (OHV’s). We classified images by date, time and bears as grizzly bears, black bears or unknown if we were unable to accurately identify species. If human recreation was present, we identified the type of recreation (truck, OHV, hiker, horse rider) and categorized the event as either motorised or non-motorised recreation.

### Modelling framework

We adapted and applied a multispecies occupancy model [9] that expands [33]’s single-species model to two or more species by assuming a multivariate Bernoulli distribution (MVB). For example, when the number of species is 2;

$$Z\;\sim \;MVB(\psi_{11},\psi_{10},\psi_{01},\psi_{00})$$ (1)

where **Z** is a 2-dimensional vector of 1’s and 0’s representing latent presence—absence of each species, and ψ_*ij*_ is the probability of each possible presence-absence combination for species *i* and *j*. ψ can be modelled within a logistic regression framework as a function of covariates. In a two-species model the natural parameters for species 1, 2 and a combination of the two are defined as:

$$f_{1}\;=\;\text{log}\;\left(\frac{\psi_{10}}{\psi_{00}}\right)\;=\;{x\prime }_{\alpha }\alpha$$ (2)

$$f_{2}\;=\;\text{log}\;(\frac{\psi_{01}}{\psi_{00}})\;=\;{x\prime }_{\beta }\beta$$ (3)

$$f_{12}\;=\;\text{log}\;(\frac{\psi_{11}\psi_{00}}{\psi_{01}\psi_{10}})\;=\;{x\prime }_{\gamma }\gamma$$ (4)

where ***x***_***α***_, ***x***_***β***_, and ***x***_***γ***_ are vectors of covariates that are predicted to explain species’ occurrence, and ***α***, ***β***, and ***γ***, are vectors of respective slope parameters. The use of probability theory allows one to test a number of hypotheses on the relationship between interacting species. For example, one might hypothesise that two species occur independently, and their probability of occurrence is solely predicted by environmental variables. Here one would want to calculate the marginal probability of occurrence for each species;

$$P(z_{1}\;=\;1)\;=\;\psi_{11}+\psi_{10}$$ (5)

$$P(z_{2}\;=\;1)\;=\;\psi_{11}+\psi_{01}$$ (6)

where *z*_*1*_ and *z*_*2*_ are the presence of species 1 and species 2 respectively. In these cases, the parameter representing conditional probability based on the presence or absence of another species (*f*_*12*_*)* is set to zero, inferring independence between species’ occurrence.

Alternatively, one could hypothesise that there is pairwise dependence between the two species i.e. their probability of occurrence is correlated. In this scenario, one would be interested in the probability of occurrence of species 1, conditional upon the presence or absence of species 2, and vice versa;

$$P(z_{1}\;=\;1\;|\;z_{2}\;=\;1)\;=\;\frac{\psi_{11}}{\psi_{11}+\;\psi_{01}}\;=\;{logit}^{-1}\;((\alpha_{0}+\;\gamma_{0})+\;\alpha_{1}x)$$ (7)

$$P(z_{1}\;=\;1\;|\;z_{2}\;=\;0)\;=\;\frac{\psi_{10}}{\psi_{10}+\;\psi_{00}}\;=\;{logit}^{-1}\;(\alpha_{0}+\;\alpha_{1}x)$$ (8)

$$P(z_{1}\;=\;0\;|\;z_{2}\;=\;1)\;=\;\frac{\psi_{01}}{\psi_{01}+\;\psi_{00}}\;=\;{logit}^{-1}\;(\beta_{0}+\;\beta_{1}x)$$ (9)

$$P(z_{2}\;=\;1\;|\;z_{1}\;=\;1)\;=\;\frac{\psi_{11}}{\psi_{11}+\;\psi_{10}}\;=\;{logit}^{-1}\;((\beta_{0}+\;\gamma_{0})+\;\beta_{1}x)$$ (10)

where γ_0_ is an intercept modifier estimated as the effect of one species on the probability of occurrence of the other.

Lastly, we may predict that although two species may occur independently, one species may influence the intensity of use at a specific site, of another species. This can be incorporated within the model by estimating two parameters, one is the intensity of use given the presence of the other species e.g. *p* (z_2_ = 1), and in the absence e.g. *p* (z_2_ = 0).

### Covariates

We built a base model for both bear species that contained covariates known to influence bear habitat use. This inclusion allowed us to control for potential habitat and landscape variables outside of our species interactions which are the main interest and focus. Distance to road and distance to stream can both influence grizzly bear and black bear habitat use, and were included as natural log transformed variables (hereby *ln*DRoad and *ln*DStream). Elevation explains variation in bear distribution, with grizzly bears usually at higher elevations relative to black bears [16,47]. Lastly, we included Normalized Difference Vegetation Index (NDVI) for the buffered area around the camera location (500m) averaged across the sampling period. NDVI positively correlates with vegetation quality [48] and forest cover and type [49], and is therefore has been used as a predictor of bear habitat use [26,50–52]. We checked for collinearity between predictor covariates, and all correlation coefficients were below 0.7. As motorised recreation is not permitted in Jasper National Park, we included a protected-area variable influencing motorised and non-motorised occurrence and intensity of use. All covariates were extracted using ArcMap (ESRI, Redlands, CA, USA).

### Species interaction effects

Grizzly bears, black bears, motorised recreationists and non-motorised recreationists were included as individual “species” within the multi-species co-occurrence model. We collapsed our data into 4-day presence-absence sampling periods, to maintain moderate probabilities of detecting all species, improving model convergence [46,53]. Cameras with less than 4 surveys were removed, leaving 182 trail camera locations for analysis. We fit a set of candidate models to test a series of hypotheses. These models varied based on, a) co-occurrence of bears and both forms of recreation (independent versus conditional), b) co-occurrence between grizzly bear and black bear, c) the effect of both forms of recreation on bear intensity of use, and d) the effect of grizzly bear occurrence on black bear intensity of use.

We fit 48 models (S1 Table) in Stan v. 2.8.0 via the Rstan (Stan Development Team, 2016) package in R [54]. Logistic prior distributions were used for all parameters [9]. We ran 3 chains each consisting of 2,000 iterations (1,000 burn-in with 1,000 sampled) and ensured model convergence by calculating Brooks-Gelman-Rubin convergence diagnostic and checking that Rhat was close to 1 [55]. Candidate models were ranked using Watanabe-Akaike Information Criterion (WAIC), which is the optimum method for contrasting fully Bayesian models [55].

### Daily activity patterns

To investigate daily activity pattern overlap between grizzly bears, black bears and motorised and non-motorised recreational activity, the timestamps of all independent events were used to build probability density functions based on the distribution of photograph count across each 24-hour period for each species. We then used these distributions to estimate the coefficient of overlapping (Δ; [56])for each pairwise relationship. To further assess the influence of interspecific interactions on daily activity patterns, we compared activity patterns for a species at a set of sites where the competing species and forms of recreational activity were present versus sites where the competing species and forms of recreational activity were absent. We used a non-parametric calculation for Δ, due to small sample sizes in some circumstances (< 75;[56]). Confidence intervals were calculated using a bootstrap method [56]. Analyses were done using the package ‘overlap’ [56,57]. We predict a high coefficient of overlapping between grizzly bears and black bears due to their similar behavior patterns. However, we expect differences to coincide with times when motorised and non-motorised recreational activity is present, with grizzly bears showing a lower amount of temporal overlap in activity with both motorised and non-motorised recreation than black bears.

## Results

Between June 15 and August 25 2014, cameras were active for 10,514 days across 182 sites. We captured 235 grizzly bear and 235 black bear observations. Of the 182 monitored sites included in the analysis, grizzly bears were photographed at 84 locations (naïve occurrence: 0.46) and black bears were photographed at 74 locations (naïve occurrence: 0.40). Grizzly bears and black bears co-occurred at 34 sites. There were 2,893 motorised recreation observations, at 73 locations (naïve occurrence: 0.40) while non-motorised recreation was more than double that of motorised, with 6,213 observations at 90 locations (naïve occurrence: 0.50). Motorised activity co-occurred more with black bears (37) than grizzly bears (27), however the inverse was true for non-motorised activity (40 black to 48 grizzly).

### Model selection

The top model had a WAIC weight of 0.88 (S1 Table). This model extended upon the base model by incorporating pairwise dependence in occurrence between bear species. Intensity of use by grizzly bears was influenced by the presence of both forms of recreational activity whereas the intensity of use by black was affected by grizzly bear presence and motorised and non-motorised recreation. The global model was ranked second with a WAIC weight of 0.11 (S1 Table). This model extended upon the top model by including co-occurrence between bear species’ and both forms of recreational activity.

### Covariates and occurrence

**Fig 2:**
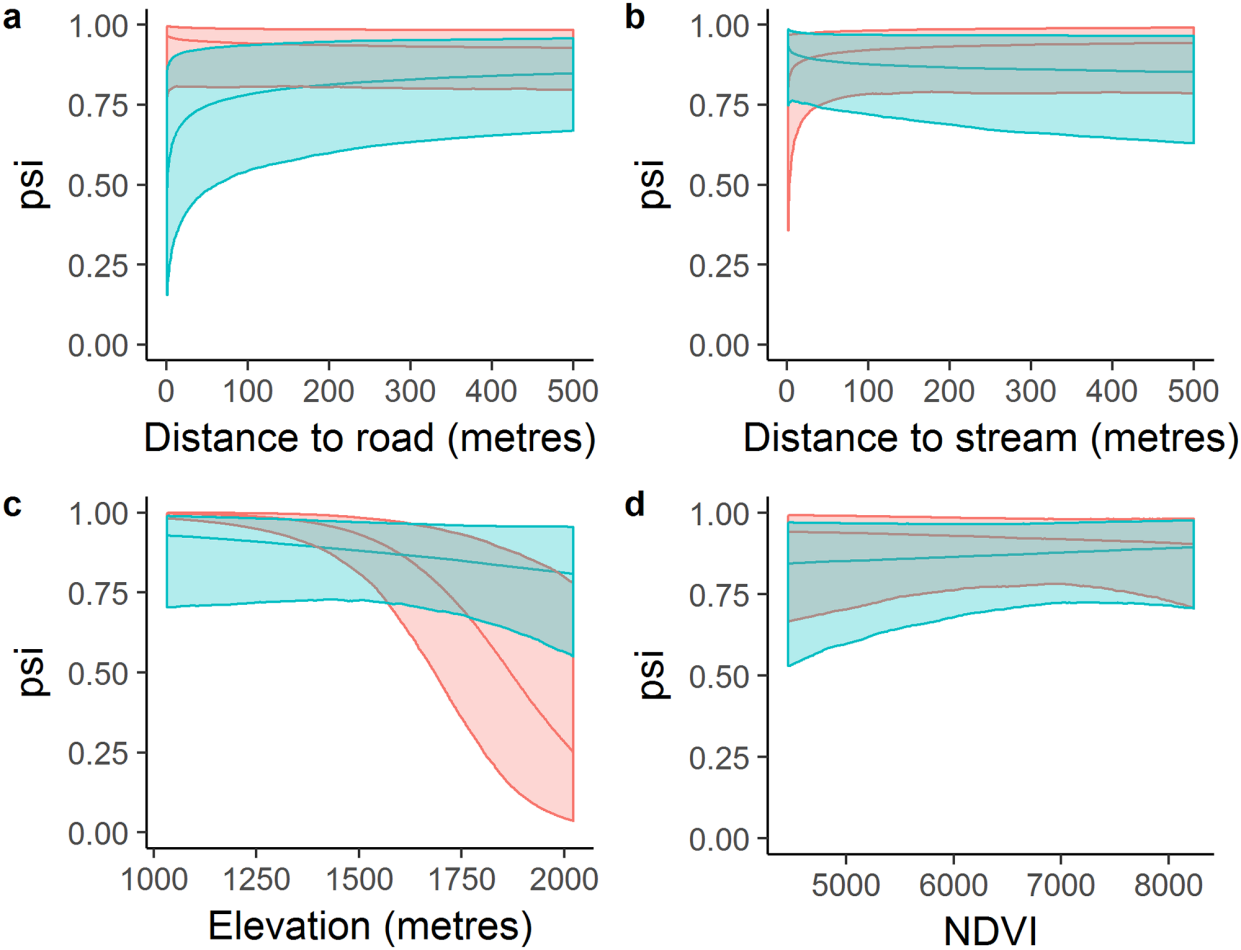
Posterior distributions of predicted relationships between probability of occurrence of grizzly bears (blue) and black bears (red) and a) DRoad, b) DStream, c) elevation and d) NDVI. Shaded areas represent the 95% credible intervals. Variables that are not included in the plot were set to their observed mean values.

We examined the direction and significance of all posterior occurrence probability distributions for landscape variables obtained from the top model (Table 1). Grizzly bear occurrence increased as distance to road increased, while black bears did not show a strong relationship with road proximity (Fig 2a). Grizzly bears occurred closer to streams, as expected (Fig 2b), with no clear difference between grizzly bear and black bear responses. Black bears displayed high probabilities of occurrence in areas of elevation less than 1500m (Fig 2c). Grizzly bears did not display a strong response to elevation, and had a much higher probability of occurrence at higher elevations (1500m–2000m) than black bears. Grizzly bears and black bears showed inverse responses to NDVI, however the influence on probability of occurrence was small and not significantly different between the two species (Fig 2d). Occurrence of motorised activity was far lower and non-motorised activity was higher in protected areas (S1 Fig). A similar pattern was observed in the intensity of use: intensity of trail use by non-motorised recreationists was higher at sites inside protected areas (S1 Fig) relative to public lands.

**Table 1:** Posterior probability estimates and 95% credible intervals for the top multi-species occurrence model. Ψ represents grizzly bear and black bear occurrence (on the logit scale) and *p* represents the intensity of use (on the logit scale). Estimates where the confidence limits overlapped zero were defined as insignificant.

	Parameter	Grizzly						Black					
		psi	upper	lower	p	upper	lower	psi	upper	lower	p	upper	lower
	Intercept	**0.61**	2.64	-1.32	**-2.09**	-1.67	-2.50	**2.40**	5.01	0.02	**-6.81**	-5.39	-8.68
Habitat	lndroad	**0.29**	0.50	0.09	-	-	-	**-0.14**	0.13	-0.40	-	-	-
	lndstream	**-0.14**	0.16	-0.44	-	-	-	**0.23**	0.67	-0.19	-	-	-
	elevation	**-0.27**	0.27	-0.80	-	-	-	**-1.76**	-0.97	-2.77	-	-	-
	NDVI	**0.11**	0.55	-0.35	-	-	-	**-0.12**	0.45	-0.71	-	-	-
Interaction	grizzly	-	-	-	-	-	-	**-2.63**	-1.30	-4.21	**4.47**	6.38	3.14
	motorised	-	-	-	**-0.67**	-0.28	-1.08	-	-	-	**-0.65**	-0.06	-1.26
	non-motorised	-	-	-	**0.49**	0.94	0.05	-	-	-	**0.82**	1.45	0.22

### Co-occurrence between species and recreational activity

**Fig 3:**
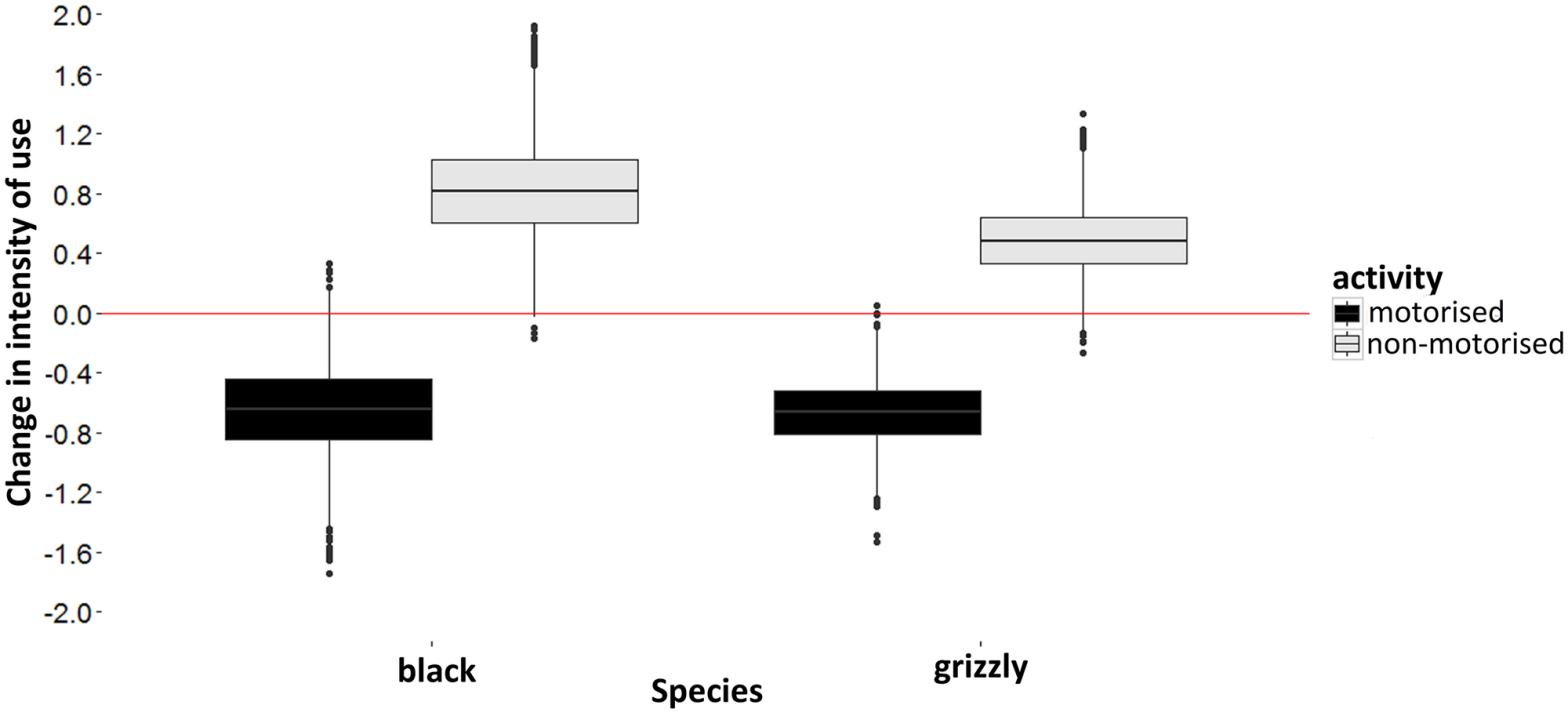
Posterior distributions for change in intensity of use by grizzly bears and black bears due to the presence of motorised (black) and non-motorised (grey) recreation (on the logit scale). The central mark represents the median, and the tails represent the 95% confidence intervals. Results were treated as non-significant if confidence intervals overlapped zero (red line).

Grizzly bears and black bears showed strong negative covariance in their occurrence, as predicted (*f*_12_ = -0.53). Interestingly, at trail locations where they co-occurred, intensity of use by black bears was higher than at sites where grizzly bears were absent (*p* (z = 1) = 0.088, *p* (z = 0) = 0.001). We found evidence of pairwise interactions between bears and recreation, however this evidence was weak as it did not significantly improve WAIC values beyond a similar model with no recreational impacts on bear occurrence (Table 1). The effect of motorised activity on the intensity of use by bears was more prominent, with grizzly bears displaying reductions in intensity of use when at sites where motorised recreation was present. Conversely, the intensity of use of black bears increased in the presence of non-motorised recreation (Fig 3).

### Activity pattern responses

**Fig 4:**
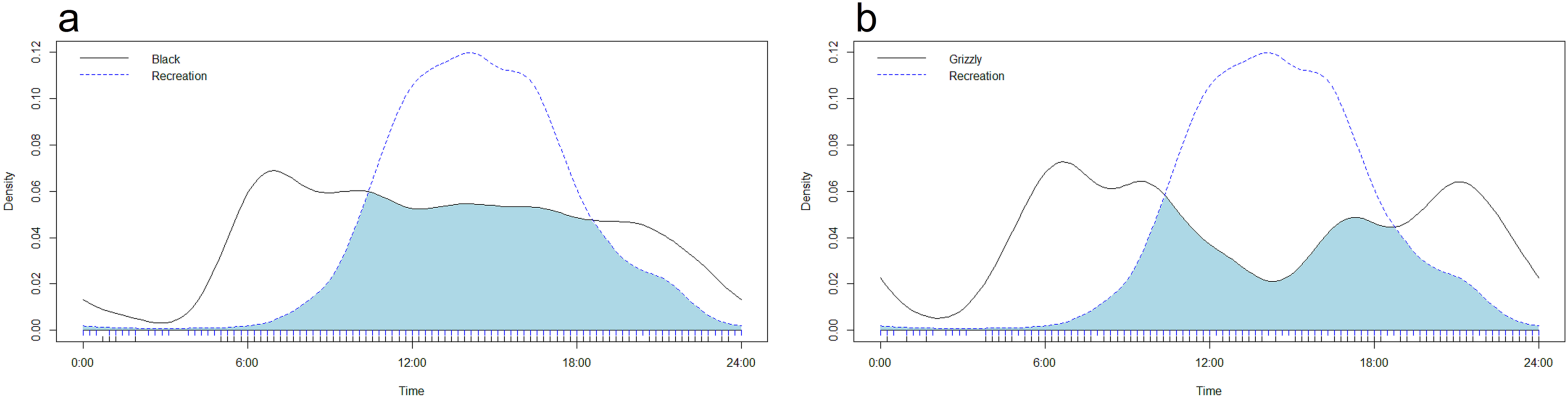
Activity overlap plots showing the relationship between time of day (hour) and the estimated kernel density distribution for independent camera events for a) recreation (blue dash) and grizzly bears (black) and b) recreation (blue dash) and black bears (black) from West-central Alberta. Blue shading represents where bear species and recreational activity temporally overlapped in terms of activity.

Daily activity patterns of grizzly bears and black bears overlapped substantially (Δ_1_ = 0.8; S2 Fig). Grizzly bears displayed a dip in activity on trails around mid-afternoon, whereas black bears had a constant level of activity throughout early to late afternoon. Black bears showed higher overlap with both forms of recreational activity than grizzly bears (Fig 4), resulting from higher levels of activity during the afternoon. Although the point estimate inferred reduced activity overlap between black bears and grizzly bears at sites where grizzly bears were present relative to sites where they were not, confidence intervals overlapped (Table 2). A similar pattern was observed between grizzly bears and motorised and non-motorised recreation (Table 2), however this difference also was not significant.

**Table 2:** Coefficient of overlap (Δ) with lower and upper confidence limits of grizzly bears and black bears with opposing bear species and recreational activity (motorised and non-motorised combined). Data were collected between June 15 and August 25 2014. Results were estimated using frequency of trail camera events per hour for each species and a combination of both recreation categories. Total number of events contributing to each Δ estimate are displayed in the n columns.

	Bear								Recreation							
	Present	lower	upper	n	Absent	lower	upper	n	Present	lower	upper	n	Absent	lower	upper	n
Grizzly	**0.87**	0.78	0.94	93	**0.84**	0.76	0.91	140	**0.41**	0.27	0.54	26	**0.52**	0.4	0.64	47
Black	**0.81**	0.72	0.89	97	**0.88**	0.84	0.94	144	**0.68**	0.58	0.78	74	**0.6**	0.48	0.71	47

## Discussion

Species occurrence is determined by biotic interactions, through competition and predation, and abiotic interactions, through landscape characteristics and habitat suitability. Novel statistical methods allow multi-species occurrence to be modelled as a function of both habitat variables and conditional upon other species’ presence [9]. Our results show that grizzly bears and black bears vary in their occurrence along trails based on surrounding landscape variables. Grizzly bears were less likely to occur close to roads; a similar response to road proximity was missing for black bears, which showed a slight increase in use of areas closer to roads. Such a result corroborates studies emphasising a disparate response to human activity between the two bear species [58,59]. Bear species occurred at different elevations, grizzly bears occurring at higher elevations than black bears. Grizzly bears are able to exploit vegetation growing at higher alpine and sub-alpine elevations such as roots of sweet vetch [13]. Higher elevations also have lower levels of human activity relative to the foothills regions, which contain a higher density of linear features, industrial activity and motorised recreation [45]. Lastly, grizzly bears and black bears showed no clear difference in occurrence relative to NDVI, inferring an absence of competitive exclusion of black bears by grizzly bears from high quality habitat [60]. The lack of a significant difference between the two species in their response to NDVI might be explained by the fact that NDVI is a poor metric for bear food quality, and is confounded by forest cover which has a high NDVI index, yet is not high food quality for bears. The use of presence-absence models [13,50] for bear foods may be a method for identifying grizzly and black bear use of habitats with different food quality.

Inclusion of pairwise dependence between grizzly bear and black bear occurrence within our top model suggests that the presence of one species affects the presence of the other. The model identified a strong negative relationship between grizzly bear and black bear co-occurrence, and this result supports research that suggests spatial segregation of the two species [61], at least within the scale of our study design. Interestingly, intensity of use by black bears was higher at locations where they co-occurred with grizzly bears. Detection probability in the traditional sense [33], when adapted for studies of free-moving animals in continuous habitats, can be influenced not only by nuisance “detection” variables, but also two metrics of interest: movement rate and abundance [35]. Our result therefore imply that black bears are either found in higher abundance when co-occurring with grizzly bears, or that they are increasing their movement on trails when co-occurring with grizzly bears, which increases their probability of being detected. The latter explanation is more likely because increased rates of displacement are common responses to predators or perceived risks [62,63]. For example, black bears increase their movement rates and home range size when sympatric with grizzly bears [61]. Our inability to identify individuals from trail camera photos made it difficult to distinguish bear abundance and individual movement behavior. Alternate studies that use non-invasive genetic sampling [64]might be able to inform us on whether this increase in intensity of use is due to a higher number of black bears, or increased movements on trails.

Our main interest was whether grizzly bears and black bears avoid areas where motorised and/or non-motorised recreational activity is present, the answer to which was not clear. Our results support work that stated grizzly bear avoidance of roads [58], however we did not find a similar response for black bears. This differential response might benefit black bears relative to grizzly bears, allowing black bears to exploit areas closer to roads due to their higher tolerance of human activity [60], especially since road are often correlated with high food quality [65]. Contrary to our predictions, model results showed that interspecific interactions had a greater impact on species occurrence than the effects of different forms of human recreational activity. Although there was no pattern in co-occurrence between either species and motorised and non-motorised recreation, we did find reduced intensity of use of trails by grizzly bears when motorised activity was present. Instead of completely avoiding trails with motorised use, grizzly bears are either found in lower abundance in the area surrounding said cameras, or they are less active on the surrounding trails. Avoidance of anthropogenic trails by wildlife has been documented previously [58], especially for trails with high human-use [6]. The increase in intensity of use of trails with non-motorised activity present could be due to a combination of factors. A diminished fear response to non-motorised activity, as well as non-motorised recreation correlating with more rugged topography (i.e. in Jasper National Park), where trails are the most efficient means of navigating those areas for both people and wildlife [25,66], would both increase the use of trails. However, identifying the true cause of this result would require further exploration.

Another way in which wildlife alter their behavior in response to competition and disturbance is by changing their daily activity patterns. Black bear activity on trails overlapped to a greater extent with motorised and non-motorised recreation than grizzly bears, which displayed more crepuscular behavior and were less active on trails during the afternoon. Small sample sizes likely influenced the ability to detect significant differences in activity patterns between sites that co-occurred with recreation and ones that did not, which makes it challenging to infer whether such a dip in bear activity is due to recreation or other factors [67] Grizzly bears did appear to show altered activity patterns when recreation was present, a behavioural change potentially aimed at reducing overlap with times of peak recreational activity, as seen in other studies [10,26], however the present result is inconclusive.

### Conclusions and management implications

Many management-based decisions are made at the single-species level, without regard for competing species that share the same landscape. This approach, although analytically far more accessible through software such as Presence [68] and the unmarked R package [69], can result in decisions with less-than-optimal outcomes for the species being managed. Multi-species occurrence models are a step forward and a tool that can be used by managers to more fully understand the system they aim to manage. Grizzly bear-black bear interactions are not generally discussed when investigating bear conservation, yet we show here that incorporating covariance between species improved model performance substantially and that the interactions between species have stronger influence on bear habitat use than human recreational activity. Our finding that grizzly bears alter the frequency of trail use in response to motorised recreation is important and can be used to inform management policy relating to recreational access. Avoidance of trails might affect grizzly bears’ ability to forage, especially if times of high recreational activity coincide with late summer and fall, when bears require high energy intake to prepare for denning. Differential response by grizzly bears and black bears to human disturbances could have implications for population demographics through risk effects, which can reduce fitness of individuals heavily investing in avoidance behaviors [28]. Restricting trail use by motorised recreationists will allow grizzly bears to maximise foraging opportunities and reduce required investment in avoidance behaviours. Lastly, future studies interested in animal habitat use should attempt to integrate spatial habitat segregation, species interactions, and the effects of human disturbance simultaneously when assessing habitat quality and making management decisions.

## Supporting information

S1 TableModel descriptions and results.WAIC weight for model *i* was calculated as $\frac{{likelihood}_{i}}{\sum {likelihood}_{i\ldots n}}$ where *n* is the total number of models.(DOCX)

S1 Fig Posterior distributions for change in occurrence (grey) and intensity of use (black) by motorised activity and non-motorised activity inside protected areas (on the logit scale). The central mark represents the median, and the tails represent the 95% confidence intervals. Results were treated as non-significant if confidence intervals overlapped zero (red line).
(TIFF)

S2 Fig Probability density functions across 24-hour period for grizzly (solid line) and black (jagged line) bears. Blue shaded area represents overlap in activity between the two species. Coefficient of overlapping was estimated at 0.8.
(TIFF)
