# Supplementary material for: The role of human outdoor recreation in shaping patterns of grizzly bear-black bear co-occurrence
Source: PLoS One. 2018 Feb 1;13(2):e0191730. doi: 10.1371/journal.pone.0191730 (PMC5794087; doi:10.1371/journal.pone.0191730)
Supplement: S1 Table — WAIC weight for model i was calculated as likelihoodi∑likelihoodi…n where n is the total number of models. (DOCX) [file pone.0191730.s001.docx]

S1 Table. Model descriptions and results. WAIC weight for model *i* was calculated as $\frac{{likelihood}_{i}}{\sum{likelihood}_{i\ldots n}}$ where *n* is the total number of models.

| Model | Species occupancy | Submodel | Detection species | Number of parameters | WAIC | Delta WAIC | Likelihood | WAIC Weight |
| --- | --- | --- | --- | --- | --- | --- | --- | --- |
| 1 | Independent | a | None | 18 | 6199.64 | 666.96 | 0.00 | 0.00 |
| 1 | Grizzly-Black | b | None | 19 | 6200.20 | 667.52 | 0.00 | 0.00 |
| 1 | Motorized | c | None | 20 | 6197.59 | 664.91 | 0.00 | 0.00 |
| 1 | Non-motorized | d | None | 20 | 6198.64 | 665.95 | 0.00 | 0.00 |
| 1 | Both recreation | e | None | 22 | 6196.54 | 663.86 | 0.00 | 0.00 |
| 1 | All | f | None | 23 | 6196.50 | 663.81 | 0.00 | 0.00 |
| 2 | Independent | a | Grizzly | 19 | 6197.25 | 664.56 | 0.00 | 0.00 |
| 2 | Grizzly-Black | b | Grizzly | 20 | 6198.64 | 665.95 | 0.00 | 0.00 |
| 2 | Motorized | c | Grizzly | 21 | 6194.62 | 661.93 | 0.00 | 0.00 |
| 2 | Non-motorized | d | Grizzly | 21 | 6195.55 | 662.86 | 0.00 | 0.00 |
| 2 | Both recreation | e | Grizzly | 23 | 6194.07 | 661.38 | 0.00 | 0.00 |
| 2 | All | f | Grizzly | 24 | 6193.59 | 660.91 | 0.00 | 0.00 |
| 3 | Independent | a | Motorized | 20 | 6184.40 | 651.72 | 0.00 | 0.00 |
| 3 | Grizzly-Black | b | Motorized | 21 | 6183.91 | 651.22 | 0.00 | 0.00 |
| 3 | Motorized | c | Motorized | 22 | 6188.68 | 655.99 | 0.00 | 0.00 |
| 3 | Non-motorized | d | Motorized | 22 | 6180.66 | 647.98 | 0.00 | 0.00 |
| 3 | Both recreation | e | Motorized | 24 | 6187.17 | 654.49 | 0.00 | 0.00 |
| 3 | All | f | Motorized | 25 | 6185.94 | 653.25 | 0.00 | 0.00 |
| 4 | Independent | a | Non- motorized | 20 | 6188.05 | 655.37 | 0.00 | 0.00 |
| 4 | Grizzly-Black | b | Non- motorized | 21 | 6188.63 | 655.95 | 0.00 | 0.00 |
| 4 | Motorized | c | Non- motorized | 22 | 6184.01 | 651.33 | 0.00 | 0.00 |
| 4 | Non-motorized | d | Non- motorized | 22 | 6190.30 | 657.62 | 0.00 | 0.00 |
| 4 | Both recreation | e | Non- motorized | 24 | 6188.37 | 655.68 | 0.00 | 0.00 |
| 4 | All | f | Non- motorized | 25 | 6190.00 | 657.32 | 0.00 | 0.00 |
| 5 | Independent | a | Both recreation | 22 | 6213.55 | 680.86 | 0.00 | 0.00 |
| 5 | Grizzly-Black | b | Both recreation | 23 | 6182.15 | 649.46 | 0.00 | 0.00 |
| 5 | Motorized | c | Both recreation | 24 | 6183.69 | 651.00 | 0.00 | 0.00 |
| 5 | Non-motorized | d | Both recreation | 24 | 6183.05 | 650.36 | 0.00 | 0.00 |
| 5 | Both recreation | e | Both recreation | 26 | 6186.98 | 654.30 | 0.00 | 0.00 |
| 5 | All | f | Both recreation | 27 | 6188.86 | 656.18 | 0.00 | 0.00 |
| 6 | Independent | a | Motorized and grizzly | 21 | 6225.39 | 692.70 | 0.00 | 0.00 |
| 6 | Grizzly-Black | b | Motorized and grizzly | 22 | 6176.29 | 643.60 | 0.00 | 0.00 |
| 6 | Motorized | c | Motorized and grizzly | 23 | 6190.63 | 657.94 | 0.00 | 0.00 |
| 6 | Non-motorized | d | Motorized and grizzly | 23 | 6179.12 | 646.43 | 0.00 | 0.00 |
| 6 | Both recreation | e | Motorized and grizzly | 25 | 6184.72 | 652.03 | 0.00 | 0.00 |
| 6 | All | f | Motorized and grizzly | 26 | 6176.12 | 643.44 | 0.00 | 0.00 |
| 7 | Independent | a | Non-motorized and grizzly | 21 | 6212.62 | 679.93 | 0.00 | 0.00 |
| 7 | Grizzly-Black | b | Non-motorized and grizzly | 22 | 6174.52 | 641.83 | 0.00 | 0.00 |
| 7 | Motorized | c | Non-motorized and grizzly | 23 | 6186.25 | 653.56 | 0.00 | 0.00 |
| 7 | Non-motorized | d | Non-motorized and grizzly | 23 | 6173.98 | 641.29 | 0.00 | 0.00 |
| 7 | Both recreation | e | Non-motorized and grizzly | 25 | 6181.07 | 648.38 | 0.00 | 0.00 |
| 7 | All | f | Non-motorized and grizzly | 26 | 6174.16 | 641.47 | 0.00 | 0.00 |
| 8 | Independent | a | Both recreation and grizzly | 23 | 5553.50 | 20.81 | 0.00 | 0.00 |
| **8** | **Grizzly-Black** | **b** | **Both recreation and grizzly** | 24 | **5532.69** | **0.00** | **1.00** | **0.88** |
| 8 | Motorized | c | Both recreation and grizzly | 25 | 5553.35 | 20.67 | 0.00 | 0.00 |
| 8 | Non-motorized | d | Both recreation and grizzly | 25 | 5556.75 | 24.06 | 0.00 | 0.00 |
| 8 | Both recreation | e | Both recreation and grizzly | 27 | 5557.14 | 24.45 | 0.00 | 0.00 |
| **8** | **All** | **f** | **Both recreation and grizzly** | 28 | **5536.71** | **4.02** | **0.13** | **0.12** |

S1 Fig 1 Posterior distributions for change in occurrence (grey) and intensity of use (black) by motorised activity and non-motorised activity inside protected areas (on the logit scale). The central mark represents the median, and the tails represent the 95% confidence intervals. Results were treated as non-significant if confidence intervals overlapped zero (red line).

Fig S2. Probability density functions across 24-hour period for grizzly (solid line) and black (jagged line) bears. Blue shaded area represents overlap in activity between the two species. Coefficient of overlapping was estimated at 0.8.
