# Supplementary figures and images for: The role of human outdoor recreation in shaping patterns of grizzly bear-black bear co-occurrence
Source: PLoS One. 2018 Feb 1;13(2):e0191730. doi: 10.1371/journal.pone.0191730 (PMC5794087; doi:10.1371/journal.pone.0191730)

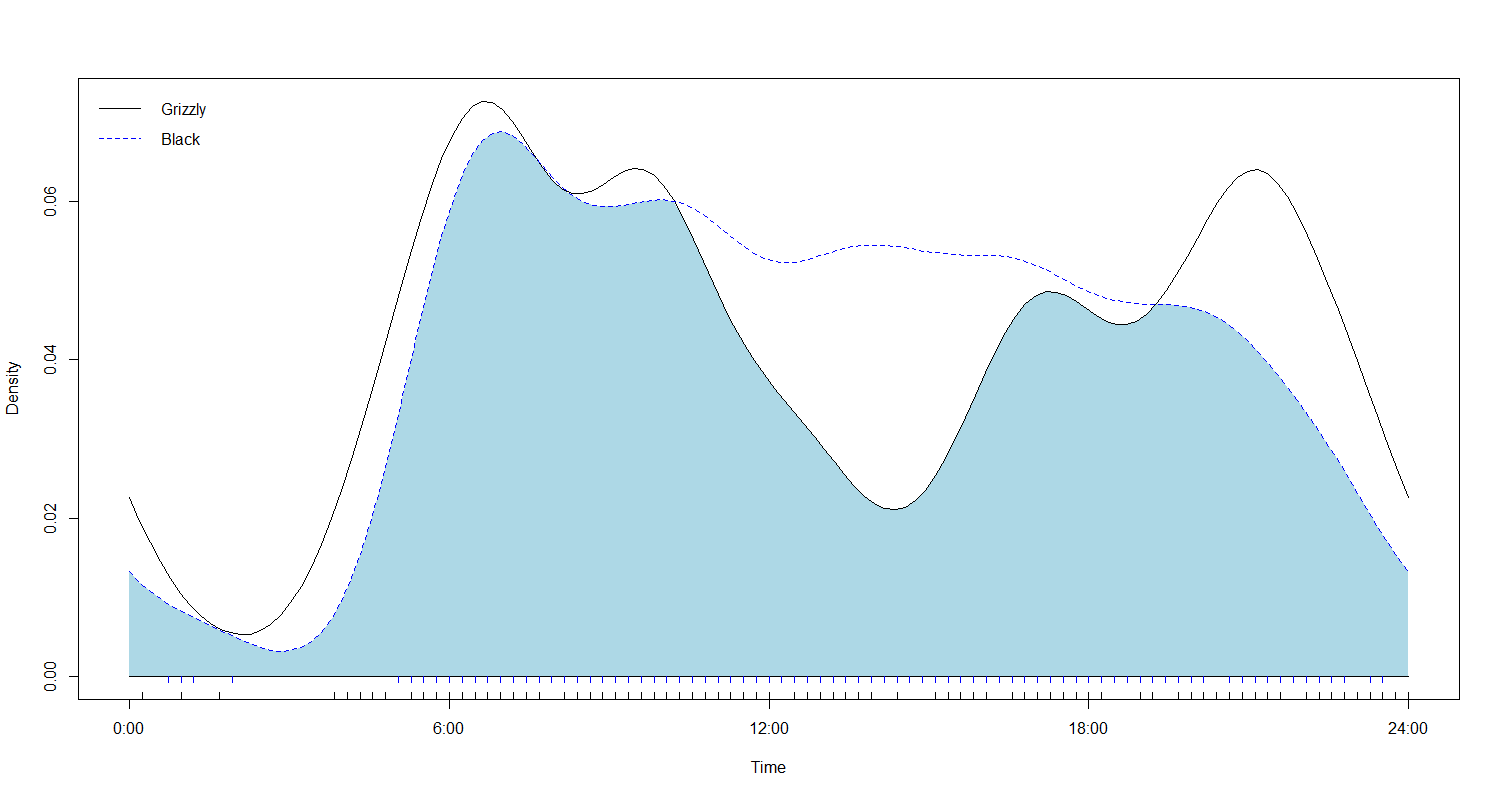

Supplement: S2 Fig — Blue shaded area represents overlap in activity between the two species. Coefficient of overlapping was estimated at 0.8. (TIFF) [file pone.0191730.s003.tiff]
